# The outcome and the risk factors of mucormycosis among patients with hematological diseases: a systematic and meta-analysis

**DOI:** 10.3389/fmed.2023.1268840

**Published:** 2023-11-30

**Authors:** Meixiao Shen, Juan Wang, Meiqing Lei, Zhiming Wang

**Affiliations:** Department of Hematology, Affiliated Haikou Hospital of Xiangya Medical College, Central South University, Haikou, China

**Keywords:** mucormycosis, hematological patients, outcome, mortality, meta-analysis

## Abstract

**Objectives:**

*Mucorale* has come into a significant pathogen over recent decades. Nonetheless, mucormycosis-related mortality rates among patients with hematological disorders remain unascertained. Thus, we conducted a meta-analysis to determine mortality rates of mucormycosis in patients with hematology-related conditions.

**Methods:**

We scoured PubMed, Embase, and Web of Science for original papers exploring the intersection of Mucormycosis and Hematological Diseases (from 2000 to 2022). We scrutinized the overall mortality across three distinct periods, as well as differentiating between high-income and middle-income nations. We further evaluated the pooled mortality and the risk differential (RD) across several subgroups.

**Results:**

The overall mortality rate for hematology patients with mucormycosis was 61%, within a 95% confidence interval (CI) of 0.54–0.68. A significant observation was that mortality rates were somewhat lower in high-income countries compared to middle-income countries (0.60 versus 0.64, *p* = 0.45). Importantly, we discovered that a combination of surgical and medical treatment significantly improved survival rates compared to medical treatment alone [mortality 0.49 versus 0.67, RD -0.19 (95%CI -0.38-0.00, I^2^ 63.7%)]. As might be expected, disseminated mucormycosis posed a significantly higher risk of death compared to isolated mucormycosis [0.60 versus 0.57, RD death 0.16 (95%CI 0.03–0.28)]. Additionally, our analysis showed no discernible differences in survival rates between genders, between patients with and without breakthrough infection, between those who received mucor-active or mucor-inactive drugs prior to mucor infection, or between those on a multi-drug regimen and those on a single drug treatment.

**Conclusion:**

Despite the high mortality rates associated with mucormycosis in patients with hematological disorders, those receiving both medical and surgical interventions, as well as those with isolated infection sites, exhibited improved survival outcomes. Conversely, factors such as gender, the presence of breakthrough infection, the use of mucor-active drugs before mucor infection, and multi-drug administration did not significantly influence patient outcomes.

## Introduction

Mucormycosis is an angioinvasive fungal disease, originating from saprophytic fungi within the *Mucorales* order (1). These ubiquitous fungi typically present as wide, ribbon-like hyphae, either aseptate or with minimal septation. Humans contract the infection chiefly through the inhalation of sporangiospores, or less frequently through ingestion of tainted food or assorted skin penetration methods (2). Over the past decades, the role of *Mucorales* as a pathogen has gained considerable prominence (3, 4). This escalation is particularly noticeable among patients with conditions like diabetes, cancer, and those undergoing solid organ or hematological transplants (4, 5). Notably, a substantial rise has been recorded in India and China, especially among patients with uncontrolled diabetes mellitus (3, 6, 7). Amidst the COVID-19 pandemic, the incidence of mucormycosis experienced a significant surge (seven cases per 1,000 patients), fifty times the previously highest recorded prevalence (0.14 cases per 1,000 patients) (8). Factors such as virus-induced endothelial dysfunction, high blood sugar levels, and immune disturbances due to corticosteroid use heightened the susceptibility to mucormycosis (9). Clinical manifestations of mucormycosis can varies based on the affected sites, with potential for rhino-orbital-cerebral, pulmonary, cutaneous, gastrointestinal, and disseminated forms (10). Diagnosis of mucormycosis primarily relies on histological evidence or positive cultures from lesion sites, due to the absence of recognized serologic markers, which can potentially cause delays in treatment initiation.

In 2005, Roden and his team conducted the first comprehensive review of mucormycosis, which included 929 cases documented from 1940 to 2003, and reported a pooled mortality rate of 44% (4). More recently, a meta-analysis by W. Jeong and his team, which scrutinized mucormycosis cases from 2000 to 2017, revealed that diabetes mellitus and hematological malignancies (42% of them being acute myeloid leukemia) were the most prevalent underlying conditions. Of the 851 patients, 389 succumbed to the diseases (11). Most available information on mucormycosis in hematology patients stems from case series and case reports. However, there has not been a meta-analysis specifically exploring the mortality rate of mucormycosis in hematological patients, primarily because of insufficiently diagnosed and documented cases. In this era marked by an increase in hematological stem cell transplantation, as well as the emergence of aggressive diagnostic techniques and new triazole drugs effective against both Aspergillus sp. and Mucorales (12, 13), the clinical characteristics and mortality rates of mucormycosis have seen a significant shift. As a result, we embarked on a meta-analysis to aggregate the mortality rates and the risk factors of mucormycosis in hematological patients spanning from January 2000 to December 2022. The protocol was registered in the INPLASY (registered number: 202370069).

## Materials and methods

This study was conducted and reported following the PRISMA guidelines (14).

### Search identification

To analyze Mucormycosis and Hematological Diseases, original articles were sought using PubMed, Embase, and Web of Science. Various combinations of keywords were utilized during the search: ‘Mucormycoses’, ‘Mucormycose’, ‘*Mucorales* Infection’, ‘*Mucorales* Infections’, ‘Zygomycoses’, ‘zygomycosis’, ‘Hematologic Disease’, ‘Blood Diseases’, ‘Blood Disease’, ‘Hematological Diseases’, ‘Hematological Disease’. The search was from January 2000 to December 2022. We conducted an additional search by examining the bibliographies of eligible studies and relevant systematic reviews (J. W. and MQ. L).

### Eligibility criteria

We established specific inclusion and exclusion standards prior to conducting our meta-analysis. For a study to be eligible, it needed to: (1) examine at least five instances of mucormycosis in patients with hematological diseases; the diagnosis of mucormycosis was according to microbiologically or pathologically, conforming to the current definition of both proven, probable, and possible cases of mucormycosis (15), only the possible cases were included in the analysis whom diagnosis was made by positive quantitative polymerase chain reaction (qPCR); (2) report the death rate, survival rate, or number of deceased and surviving patients; and (3) be published as a full paper in English. If multiple publications included the same group of patients, we opted for the most comprehensive or informative study for our analysis. Studies were excluded if they presented fewer than five mucormycosis cases in hematological patients or relied on autopsy results for mucormycosis evidence (either microbiological or pathological). Animal studies and non-English language studies were also not considered for inclusion.

### Data extraction

Two researchers (MX. S. and J. W.) individually reviewed and extracted data from all potentially relevant papers, with any discrepancies resolved through discussion. A third researcher (ZM. W.) adjudicated any contentious points. Our meta-analysis followed the PRISMA guidelines to maintain quality (14). Data retrieved encompassed author, publication year, population country, study duration, gender, age, follow-up period, number of deaths and total cases of hematological patients diagnosed with mucormycosis, underlying hematological disease, infection sites, and treatment strategy. We also extracted mortality data related to specific groups such as gender, proven, probable and possible mucormycosis, cases received surgery or not, disseminated infection and isolated infection, cases with or without breakthrough invasive fungal infection (IFI), and whether a mucor-active or mucor-inactive drug was administered prior to infection. A few studies provided only mortality data without separate detailed information. Breakthrough IFIs, defined as any IFI occurring during treatment with an antifungal drug (as prophylaxis, pre-emptive or targeted therapy), including those inside and outside the agent’s spectrum of activity (16). Mucor active drugs included AmB-based drugs, posaconazole and isavuconazole. The other antifugal drugs without clear antimucor spectrum were classified as mucor inactive drugs.

### Quality assessment of the studies

We employed the Newcastle-Ottawa Scale (NOS) for quality assessment of the studies included (17). This scale evaluates three components - study selection (0–4 points), comparability (0–2 points), and outcome (0–3 points), yielding a maximum score of 9, where a higher score indicates superior study quality.

### Outcomes

Our primary aim was to evaluate the aggregate mortality in hematological patients with mucormycosis. Mortality was compared across three study periods (2000 to 2009, 2009 to 2015, 2016 to 2022) based on publication year and two income groups (high and middle) using World Bank 2022 data (no data from low-income countries were available). Secondary outcomes, which could not be extracted from each study, involved comparisons of mortality due to mucormycosis: (a) between male and female hematological patients; (b) comparing patients who received combined medical-surgical therapy to those who received medical therapy alone; (c) between patients experiencing disseminated infection and those with isolated infection; (d) between patients who had a breakthrough infection and those who did not; (e) between patients who underwent combined multi-drug therapy and those who were on single-drug therapy; (f) between hematological patients taking mucor-inactive drugs and those on mucor-active drugs for prophylaxis or treatment before developing mucormycosis infection.

### Subgroup analysis

We conducted subgroup analyses on the following: (a) mortality rates among patients with proven, probable, and possible mucormycosis. (b) mortality rates among patients with different underlying diseases, including acute myeloid leukemia, acute lymphocytic leukemia, lymphoma, myelodysplasia syndrome, aplastic anemia, multiple myeloma, chronic lymphocytic leukemia, and chronic myeloid leukemia.

### Statistical analysis

To aggregate the survival outcomes quantitatively, we applied a random-effects model to determine the collective mortality rate of mucormycosis infection in hematological diseases, given significant heterogeneity where I^2^ > 50%. In the absence of such heterogeneity, we utilized a fixed-effect model. Mortality variations in predetermined subgroups were evaluated using the χ2 test. We conducted publication bias assessment using a funnel plot accompanied by Begger’s and Egger’s tests. A sensitivity analysis was also implemented to examine the impact of outcomes from these qualifying studies. The pooled mortality of each group, with a 95% confidence interval (CI), were reported, and also the risk differential (RD) of mortality for comparisons among various groups: male versus female patients, those receiving combined medical-surgical therapy versus medical management alone, multi-drug versus single-drug treatment recipients, patients with disseminated infection versus localized infection, individuals with breakthrough infection versus those without, and patients administered mucor-inactive versus mucor-active drugs for prophylaxis or pre-infection treatment. We considered a *value of p* of less than 0.05 as statistically significant in all two-sided statistical tests. We conducted all analyses using Stata Statistical Software (version 15.0, Stata Corp., College Station, TX, United States).

## Result

**Figure 1:**
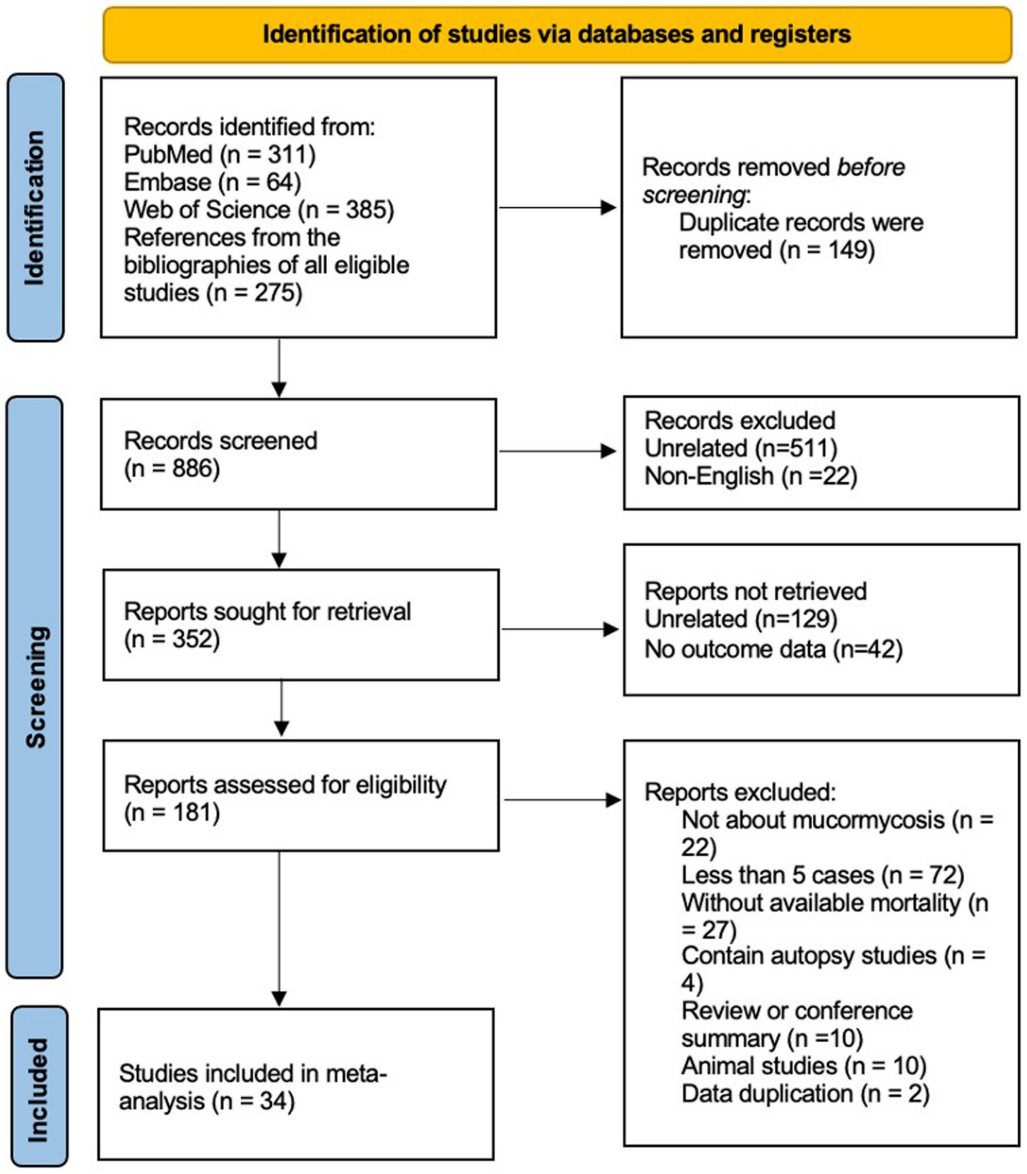
Search strategy and the process of the systematic review.

In the identification of relevant studies, an initial search led us to a total of 1,035 potential studies (Figure 1). After eliminating 149 duplicate entries, we screened the remaining records, resulting in the exclusion of 534 studies (511 studies were unrelated about hematological patients and mucormycosis, the other 22 studies were not published in English). Out of the remaining 352 studies, we found 181 suitable for retrieval (129 studies were unrelated, and no outcome data were extracted from the other 42 studies). However, upon further assessment, we determined 22 of these studies were not directly associated with mucormycosis, while 72 reported fewer than 5 cases. Additionally, 27 studies lacked sufficient data to estimate mortality, and 4 were based on autopsy findings. 10 reviews or conference summaries, along with another 10 animal-based studies, were also eliminated. We identified 2 studies with overlapping data. Ultimately, our meta-analysis incorporated 34 articles, comprising a total of 811 patients. Out of these patients, 485 succumbed to diseases. The clinical data from these selected studies can be found in the Supplementary Table S1. The median NOS score, available in Supplementary Table S2, was 6, with a range of 5 to 9.

### Pooled mortality of mucormycosis in hematology patients

**Figure 2:**
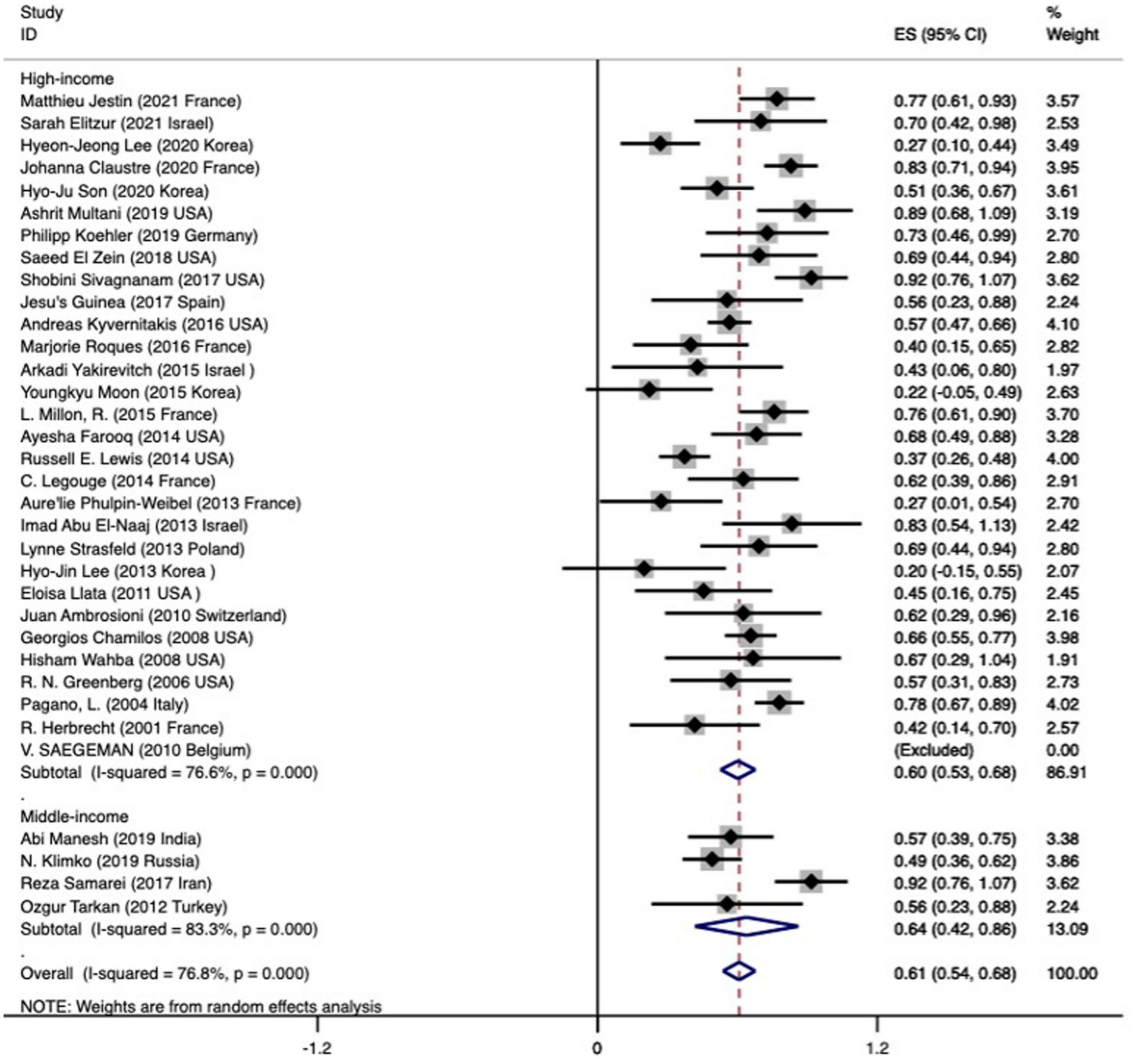
Forest plot showing the overall pooled mortality of subjects with mucormycosis among hematologic patients by across two subgroups based on the income of the countries. The mortality in the included studies is represented by the black square with horizontal bars indicating the 95% confidence interval. The diamond at the end of each subgroup and overall denotes the pooled mortality in each subgroup and the overall pooled mortality, respectively.

The overall mortality rate (95% CI) was 0.61 (0.54–0.68) (Figure 2). The analysis of mortality showed significant heterogeneity (I^2^ 76.8%). We put these data into three groups according to the articles published year, 2000–2009, 2010–2015, and 2016–2021 (Table 1). However, our data did not indicate that the mortality significantly improved over the years (0.66 versus 0.53 versus 0.66, *p* = 0.019, further proved that only 2000–2009 and 2010–2015 has significantly different). We found the pooled mortality rates of 0.60 (95%CI 0.53–0.68, I^2^ 76.6%) versus 0.64 (0.42–0.86, I^2^ 83.3%) for high-income countries verse middle-income countries, respectively (Figure 2 and Table 1). There was a trend seemed that the mortality in high-income countries was lower than those in middle-income countries, while did not reach significantly (*p* = 0.45).

**Table 1:** Pooled mortality in mucormycosis among hematologic patients and the various subgroup of subjects.

	Number of studies	Number of dead/ Number of subjects	Pooled mortality (95% CI)	I^2^ value	*p*
All studies	34	485/811	0.61 (0.54–0.68)	76.8%	
Classification based on the income of the countries^a^					0.45
Low-income countries	–	–	–	–	–
Middle-income countries	4	61/108	0.64 (0.42–0.86)	83.3%	
High-income countries	30	424/703	0.60 (0.53–0.68)	77.6%	
Classification based on the published year					0.019
2001–2009#	5	109/161	0.66 (0.54–0.77)	47.4%	
2010–2015#	14	125/234	0.53 (0.41–0.64)	68.9%	
2016–2022	15	251/416	0.66 (0.55–0.76)	81.4%	
The different definition of mucormycosis					0.114
Proven	10	146/222	0.69 (0.60–0.78)	48%	
Probable	5	52/85	0.64 (0.46–0.82)	67.7%	
Possible	2	11/12	0.86 (0.60–1.12)	100.0%	
Underlying disease					0.102
AML	19	72/111	0.62 (0.53–0.72)	0.0%	
ALL	17	43/70	0.66 (0.54–0.78)	0.0%	
Lymphoma	14	40/47	0.83 (0.68–0.97)	0.0%	
MDS	8	14/17	Incalculable	Incalculable	
AA	7	8/10	0.75 (0.33–1.17)	100%	
MM	4	3/5	Incalculable	Incalculable	
CLL	5	3/6	0.50 (−0.19–1.19)	100%	
CML	5	4/7	Incalculable	Incalculable	

CI, confidence interval; AML, acute myeloid leukemia; ALL, acute lymphocytic leukemia; MDS, myelodysplasia syndrome; AA, anemia aplastic; MM, multiple myeloma; CLL, chronic lymphocytic leukemia; CML, chronic myeloid leukemia.

a Classification based on the World Bank data (2022), #: *p* 0.005, −: not reported.

The mortality rates between different groups of mucormycosis patients were compared. The mortality rates showed no significant difference between the group with proven mucormycosis (0.69, 95% CI 0.60–0.78) and the group with probable mucormycosis (0.64, 95% CI 0.46–0.82), as well as between the group with proven mucormycosis and the group with possible mucormycosis (diagnosed by positive qPCR) (0.86, 95% CI 0.60–1.12). However, there was no significant difference (*p* = 0.114) between these three groups. Among mucormycosis patients, the most common hematological diseases were acute myeloid leukemia (AML), acute lymphocytic leukemia (ALL), and lymphoma. The pooled mortality rates for these diseases were as follows: 0.62 (95% CI 0.53–0.72) for AML, 0.66 (95% CI 0.54–0.78) for ALL, and 0.83 (95% CI 0.68–0.97) for lymphoma. In all three cases, there was no heterogeneity (I^2^ = 0%). The mortality rates for other underlying diseases are presented in Table 1, and no significant differences were found among them (*p* = 0.102).

### Analysis of publication bias

**Figure 3:**
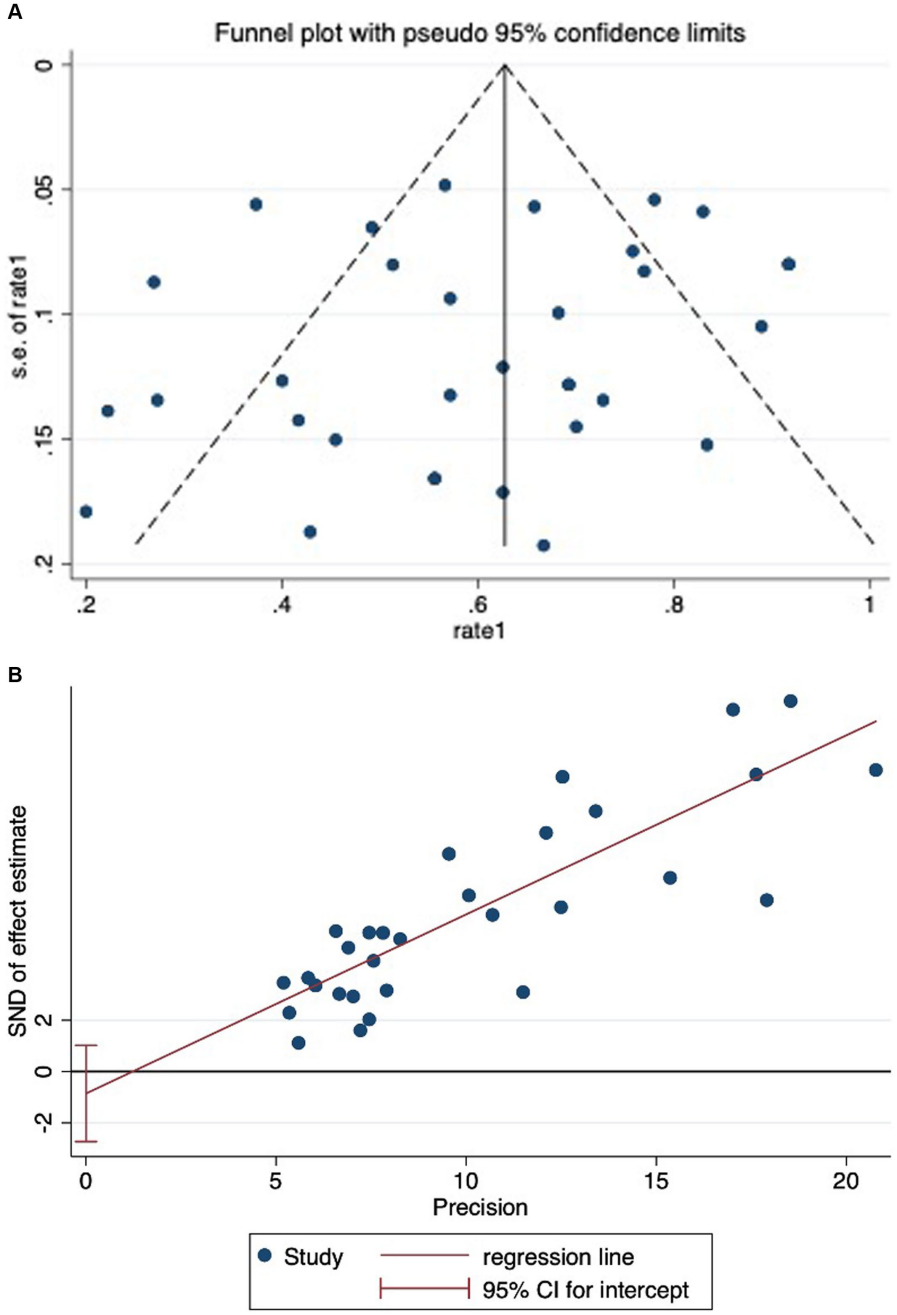
**(A)** Funnel plot showing significant publication bias in studies reporting on mortality in mucormycosis among hematologic patients. **(B)** Egger’s test showing no significant publication bias in studies reporting on mortality in mucormycosis among hematologic patients.

Significant bias was noted upon examining the funnel plot (refer Figures 3A,B). Nonetheless, the outcome of statistical tests did not corroborate the presence of substantial bias (Begg’s test: *p* = 0.209; Egger bias: *p* = 0.359).

### Examination of sensitivity

A sensitivity analysis scrutinizes the impact of individual studies on the overall estimate of the meta-analysis. Here, the pooled mortality was recalculated excluding each study one by one. The analysis thus determines the robustness and stability of the studies. In our case, the data for mortality rates in hematology patients with mucormycosis proved consistent (refer Supplementary Figure S1).

### Assessing mortality and RD of mortality among various patient groups

#### Male vs. female comparison

Out of 21 studies (n = 515) that yielded necessary data, pooled mortality rates were determined as 0.63 (95%CI 0.54–0.72, I^2^ 58.6%) for males and 0.60 (0.49–0.72, I^2^ 73.7%) for females. The RD of death displayed no significant difference (RD -0.04 (95%CI -0.12 to 0.04, I^2^ 0%)) when comparing male and female hematology patients with mucormycosis (Supplementary Figure S2). An examination of these 18 studies did not uncover any publication bias (*p* = 0.976 for Begg’s test; *p* = 0.320 for Egger bias) (refer Table 2).

**Table 2:** The subgroup analysis of pooled mortality in mucormycosis among hematologic patients.

	Pooled mortality (95% CI)	I^2^ value	RD (95% CI)	I^2^ value
Combined medical-surgical therapy	0.49 (0.34–0.63)	78.4%	-0.19 (-0.38–0.00)	63.7%
Sole medical therapy	0.67 (0.60–0.74)	1.4%		
Dissemination infection	0.60 (0.43–0.77)	64.7%	0.16 (0.03–0.28)	20.3%
Isolated infection	0.57 (0.47–0.68)	68.6%		
Male	0.63 (0.54–0.72)	58.6%	-0.04 (-0.12–0.04)	0.0%
Female	0.60 (0.49–0.72)	73.7%		
Breakthrough infection	0.63 (0.53–0.74)	67.9%	0.06 (-0.07–0.19)	18.4%
Without breakthrough infection	0.51 (0.30–0.72)	79.0%		
Multi-drug therapy	0.49 (0.37–0.61)	27.8%	-0.11 (-0.33–0.11)	59.7%
Single-drug therapy	0.61 (0.45–0.77)	59.6%		
Mucor-inactive drugs prior to mucor infection	0.70 (0.56–0.84)	70.5%	0.06 (-0.34–0.46)	79.0%
Mucor-active drugs prior to mucor infection	Incalculable	Incalculable		

CI, confidence interval; RD, risk differential.

#### Contrasting mortality and RD between combined medical-surgical therapy and sole medical therapy

**Figure 4:**
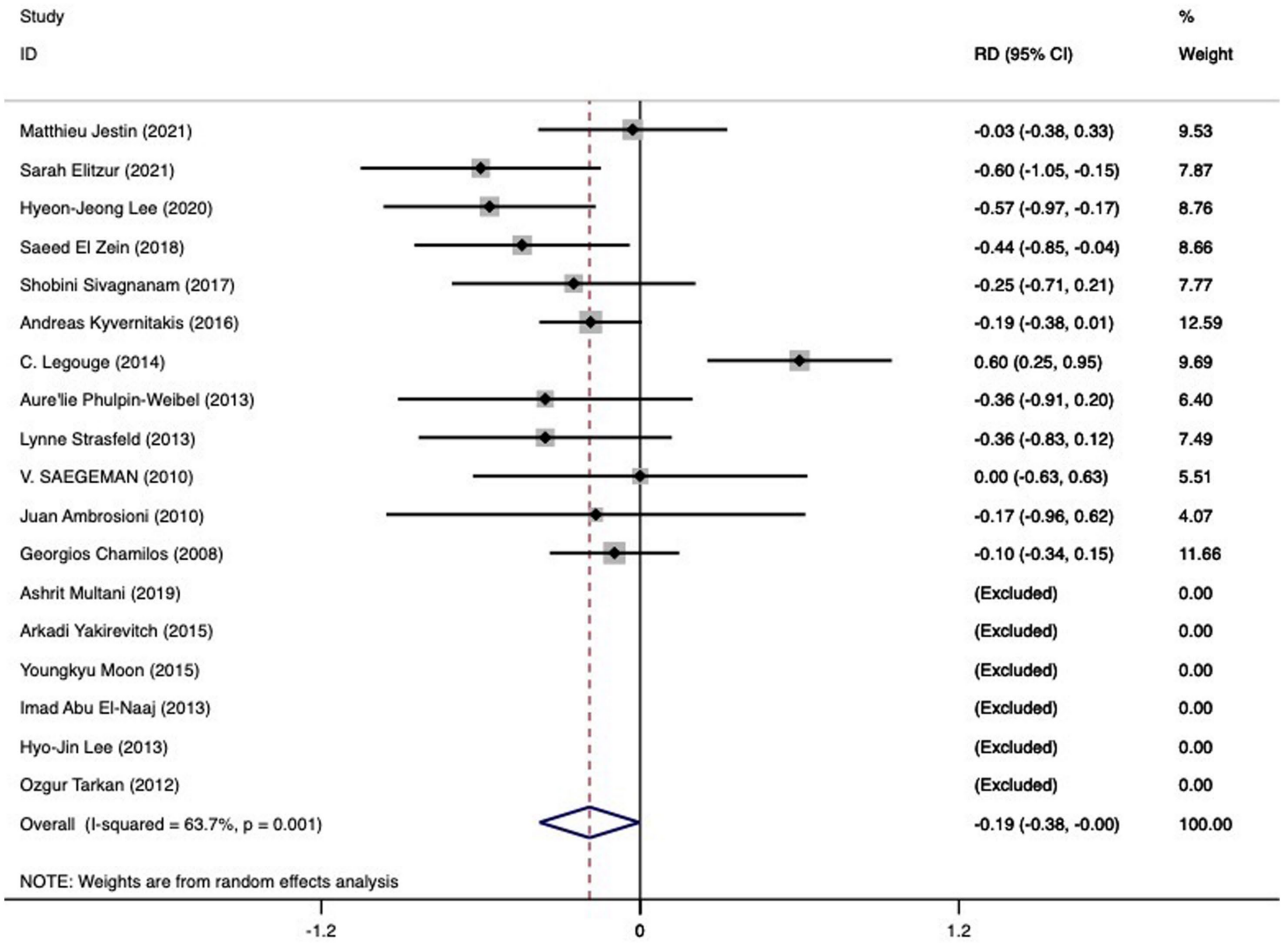
The RD of death in hematologic patients with mucormycosis undergoing combined medical-surgical therapy (left side of the solid vertical line) versus sole medical therapy (right side of the solid vertical line). The individual RD is represented by grey squares, while a diamond indicates summary RD. The horizontal lines across the squares show the 95% confidence interval.

Among the 18 studies (n = 360) yielding relevant data, pooled mortality rates were found to be 0.49 (95%CI 0.34-0.63, I^2^ 78.4%) for the combined therapy and 0.67 (95%CI 0.60-0.74, I^2^ 1.4%) for medical therapy alone. An observed pooled RD of -0.19 (95%CI -0.38 to -0.00, I^2^ 63.7%) pointed towards the favor of combined medical-surgical therapy for patients with mucormycosis in the hematological category (refer Figure 4; Table 2). The examination of these 18 studies did not reveal any notable publication bias (*p* = 0.837 for Begg’s test; *p* = 0.586 for Egger bias).

#### Comparison of mortality and RD of disseminated mucormycosis versus isolated mucormycosis

**Figure 5:**
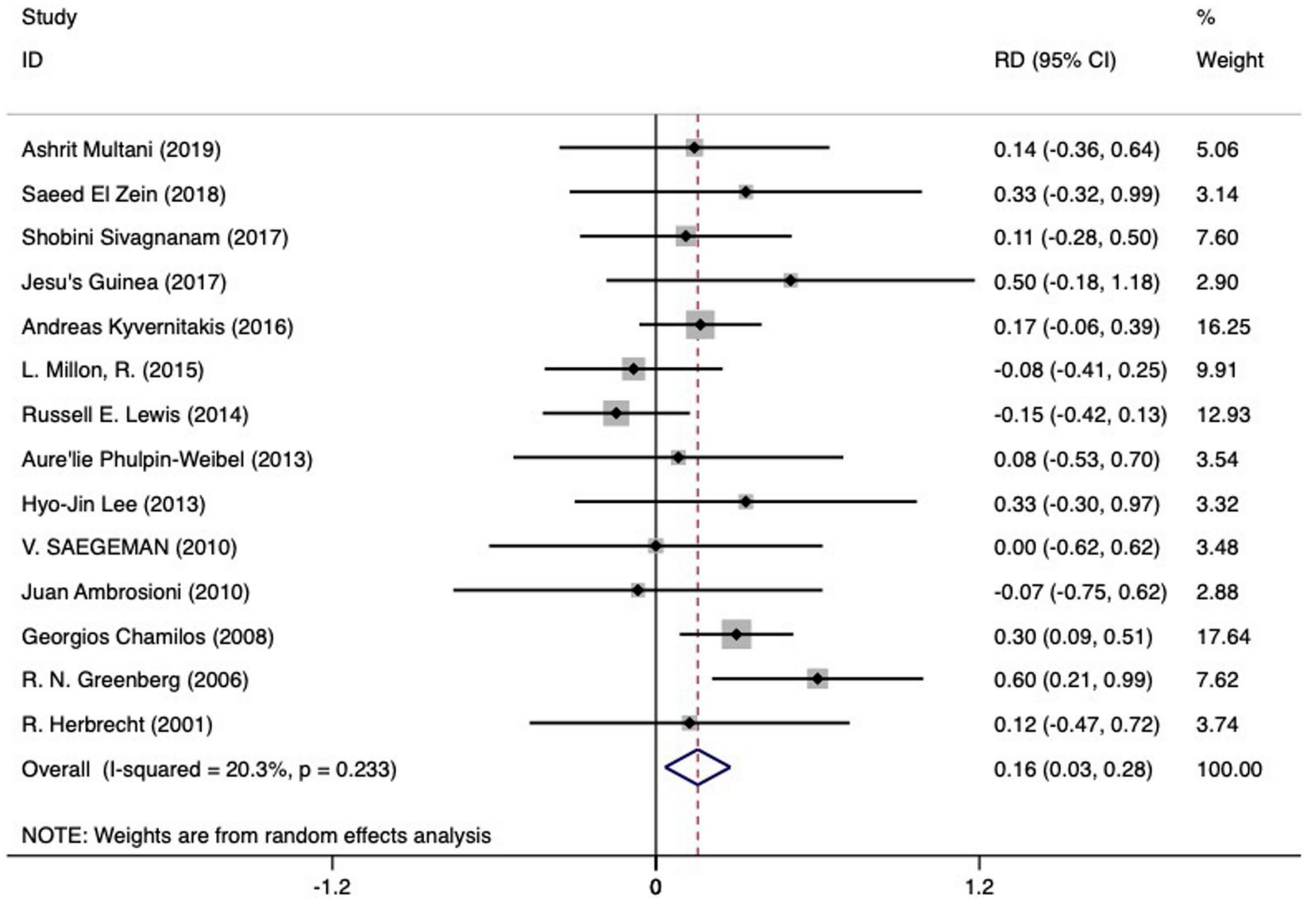
The RD of death in hematologic patients with mucormycosis undergoing dissemination infection (left side of the solid vertical line) versus isolated infection (right side of the solid vertical line). The individual RD is represented by grey squares, while a diamond indicates summary RD. The horizontal lines across the squares show the 95% confidence interval.

Available data enabled the comparison of mortality rates between disseminated mucormycosis (90 subjects) and isolated mucormycosis (321 subjects). A pooled mortality rate of 0.60 (95% CI 0.43–0.77, I^2^ 64.7%) was noted for the former, while the latter saw rates of 0.57 (95% CI 0.47–0.68, I^2^ 68.6%). As indicated in Figure 5 and Table 2, a higher RD of death (0.16 (95%CI 0.03 to 0.28)) was associated with disseminated mucormycosis compared to isolated mucormycosis. Significant heterogeneity was observed (I^2^ 20.3%), yet no significant bias was discerned (Begg’s test: *p* = 1.000; Egger bias: *p* = 0.877).

#### Comparison of mortality and RD of breakthrough infection versus not breakthrough infection

An analysis of 11 studies (n = 424) explored the mortality rates in patients with mucormycosis, both with and without breakthrough infection. Mortality rates were observed to be 0.63 (95%CI 0.53–0.74) for those with breakthrough infection and 0.51 (95%CI 0.30–0.72) for those without. No significant difference was identified in the RD of death (0.06, 95%CI -0.07 to 0.19) (refer Supplementary Figure S3; Table 2). Heterogeneity was found to be non-significant (I^2^ 18.4%). Furthermore, no publication bias was detected among these 11 studies (Begg’s test value of p: 0.640; Egger bias value of p: 0.406).

#### Comparison of mortality and RD of multi-drug treatment versus single drug

An examination of 13 studies (*n* = 226) was performed to assess mucormycosis treatment outcomes with multi-drug and single-drug therapies. Mortality rates for multi-drug treatment were 0.49 (95%CI 0.37–0.61, I^2^ 27.8%), while single-drug therapy exhibited mortality rates of 0.61 (95%CI 0.45–0.77, I^2^ 59.6%). There was no significant difference noted in the RD of death (-0.11, 95%CI -0.33 to 0.11) (refer Supplementary Figure S4; Table 2). Although there was significant heterogeneity detected (I^2^ 59.7%), publication bias was not present (Begg’s test: *p* 0.721; Egger bias: *p* 0.689).

#### Comparison of mortality and RD of taking mucor-inactive drugs and those on mucor-active drugs before developing mucormycosis infection

We analyzed 8 studies (*n* = 188) that reported on hematological patients who received either mucor-active drugs (7/10) or mucor-inactive drugs (106/178) prior to encountering breakthrough mucormycosis. The group administered with mucor-active drugs presented a pooled mortality rate of 0.70 (95%CI 0.56–0.84). For the mucor-inactive drug group, there was an insufficient data pool for conducting a meta-analysis. The death RD between the two groups did not exhibit a significant disparity (0.06, 95%CI -0.34 to 0.46) as depicted in Supplementary Figure S5 and Table 2. Notably, a high level of heterogeneity (I^2^ 79%) was detected, yet no publication bias was found (*p* = 0.452 for Begg’s test; *p* = 0.222 for Egger bias).

## Discussion

Our extensive systematic review offers a contemporary exploration of mucormycosis cases in hematological patients, shedding light on the impacts of this rare infection. A notably high mortality rate of 61% was found, which exceeds the 41% noted by Jeong et al.’s study (18) that incorporated a variety of underlying diseases. While the pooled mortality rate was somewhat lower in high-income countries compared to middle-income countries, the difference wasn’t significant. Our findings did not suggest any significant improvement in mortality rates over time. Remarkably, outcomes were significantly better for those treated with a combination of medical and surgical therapies, compared to those receiving medical treatment alone (mortality rates: 0.49 vs. 0.67). As one might expect, disseminated mucormycosis carried a higher death risk compared to isolated mucormycosis. Survival rates appeared to be similar across genders, those with or without breakthrough infection, those administered with mucor-active or mucor-inactive drugs prior to infection, and in both multi-drug and single-drug groups.

In both Europe and the United States, hematological malignancies emerged as the most frequent predisposing condition for mucormycosis. Our findings reveal that the susceptibility to mucormycosis is heightened in patients diagnosed with AML, ALL, myelodysplastic syndrome, aplastic anemia, and lymphoma. Particularly during the neutropenic phase, these patients’ immune function can decrease, and the infection can diffuse, result in delaying chemotherapy schedules. Yet, when the primary disease enters remission, the potential for mucormycosis recovery or elimination could emerge. Consequently, the strategy for managing mucormycosis in hematological patients largely should be early diagnosis, combined surgical treatment with systemic antifungal treatment, and opting for a less intense chemotherapy regimen or one that causes mild myelosuppression could prove to be a beneficial course of action. Anti-mucor regimen was follow the guideline of the European Confederation of Medical Mycology and the Mycoses Study Group Education and Research Consortium (19).

Challenges in promptly diagnosing and initiating treatment, coupled with intricate underlying conditions such as immunosuppression, could be contributing factors to the high mortality observed in these patient groups (20). Multiple studies have attempted to examine the death rate. A pair of prospective investigations specific to mucormycosis documented mortality rates of 41 and 47% (21, 22) respectively. However, these rates differed according to the site of the infection: a soaring 96% in disseminated cases, 85% in gastrointestinal instances, and 76% in pulmonary infections (4). However, there is no study reported the pooled mortality in hematology patients. With the advent of the new kinds of triazole drugs, the survival was improved over time in Mushu’s research (about pulmonary mucormycosis), especially in high-income countries (23), which is consistent with our research. In high-income area, physicians might have more access to diagnosis mucor as a pathogen timely and new anti-mucor drugs were accessible, which may potentially contribute to relatively better outcomes. In relative low-income area, the rate of effective drug use was quite low since patients were not affordable for the high price in mucor-active drug. In the present research, our data did not indicate that the mortality significantly improved over the years. This result may cause by the proportion of ICU patients were high in the included research (24, 25), which indicated a worse outcome certainly. And it also had the other influent factors might affect this trend.

Echoing previous observations, significantly improved survival rates were noted when surgical intervention was paired with medical treatment, in comparison to relying on medical treatment alone. The optimal recovery pathway seems to be a blend of liposomal amphotericin B and surgical intervention (21). This survival advantage associated with a surgical-medical blend has been demonstrated in other studies as well (4, 20, 21, 26).

Although the benefits of debridement appear evident, the decision to proceed with surgery is often dependent on the patient’s performance status and the primary disease’s state of remission, which could introduce bias (26, 27). Understandably, patients in a generally weak condition tend to opt for conservative medical treatment. Otherwise, the infection site could also be a factor. For instance, the advantages of pulmonary resection in patients with multifocal or disseminated mucormycosis are uncertain (26). While surgical treatment can reduce patient mortality by 55%, certain restrictions apply for thrombocytopenic patients (28). Consequently, the European Confederation of Medical Mycology and the Mycoses Study Group Education and Research Consortium endorse early comprehensive surgical treatment for mucormycosis whenever feasible, along with systemic antifungal treatment (19).

Medical practitioners may overlook early, nonspecific clinical signs, which could lead to delayed diagnosis and ultimately, fatal disseminated infections (29). Both solid organ transplant recipients and patients with hematological malignancies face a heightened risk of disseminated mucormycosis (11). Widespread disease following angioinvasion is the most dreaded manifestation of mucormycosis due to its high mortality rate. Past studies have recorded mortality rates ranging from 63.9 to 96% for disseminated mucormycosis (4, 6, 11). In contrast, localized mucormycosis, with an outcome odds ratio (OR) of 0.06 and a 95% CI between 0.01 and 0.6 (*p =* 0.019) (30), is linked to more favorable patient outcomes. The restrictive antifungal effect may be attributable to multiple infection sites and the differential drug permeability across various affected locations.

It’s worth noting that we discovered no significant difference in mortality rates between groups receiving either multi-drug or single-drug treatment. A recent study corroborated our findings, showing no significant variance in 6-week mortality rates when comparing initial combination therapy to monotherapy for mucormycosis treatment in adults with hematologic malignancies (43% vs. 41%, *p* = 0.85) (30). An even more recent propensity score analysis involving 106 mucormycosis patients with hematological malignancies indicated no survival benefit for those starting on a combination antifungal regimen including i.v. liposomal-amphotericin B (AmB), posaconazole, and an echinocandin (OR = 0.8, 95%CI 0.3–2.4; *p* = 0.69) (30). Rajeev Soman and colleagues postulated that the outcomes of posaconazole or isavuconazole monotherapy were on par with amphotericin B treatment. Even in COVID-19-associated mycormycosis (31), the Vital trial further echoed this sentiment by stating that isavuconazole’s clinical efficacy and tolerability were comparable to AmB’s in mucormycosis cases (mortality 33.3% vs. 41.3%; *p* = 0.60). Mucormycosis treatment guidelines strongly support liposomal AmB 5–10 mg/kg per day as the first-line therapy, with isavuconazole and posaconzole carrying moderate and slight recommendations, respectively (10). Still, some prospective research involving small samples suggested that combination antifungal therapy (echinocandins + AmB) yielded promising results (32). We could not rule out the possibility that these combination therapies were implemented in more severe diseases with a worse prognosis. Future studies should investigate deeper into the antagonism or synergism between different anti-fungal drugs and whether AmB holds any incremental benefits over azoles at all.

Incidence rates for IFI could reach up to 5–8% following Hematopoietic Stem Cell Transplantation (HCT) (33). Such infections are particularly prevalent in patients under prophylactic, pre-emptive, and targeted antifungal therapy, especially those with immunodeficiency (34–36). An individual’s overall state of immunosuppression plays a vital role in determining their infection risk (33). Pedro Puerta-Alcalde found that invasive aspergillosis (45.5%) was the most common pathogen in breakthrough IFIs among hematological malignancy patients, followed by candidemia (19%) and mucormycosis (5.8%) (36). In this extensive, prospective, multi-center Spanish study on hematological malignancy patients with breakthrough IFI, the 100-day mortality rate was 47.1%, with breakthrough IFI was the contributing cause to death in 61.4% of these cases (36). Jin Yeong Hong et al. revealed that patients with myeloid malignancy receiving posaconazole tablet prophylaxis experienced a higher mortality rate (30.0%) in breakthrough IFI cases compared to non-IFI patients (1.9%; *p* < 0.001) (37). However, the high mortality rate associated with mucormycosis following voriconazole exposure mirrors that seen in patients without prior voriconazole use (38). Further examination of breakthrough IFIs and their outcomes is crucial.

Early diagnosis is crucial in preventing tissue invasion and dissemination, although it can be challenging due to the often-nonspecific symptoms that are typically associated with other infections. This complexity is further compounded by the limited awareness of pathogen identification among clinicians and the inadequate sophistication of reference laboratories and mycology technologists, which may contribute to the difficulty in timely diagnosis. However, despite these obstacles, histopathology, direct examination, and culture remain essential diagnostic tools. Furthermore, promising advancements have been made in the field of mucormycosis diagnosis. Methods focused on detecting *Mucorales* DNA in blood have shown potential, offering the possibility of quicker and earlier diagnosis (1, 39).

The case studies for this review were sourced from an exhaustive database search employing a systematic strategy, thereby enhancing the solidity of the evidence. There is an absence of publication bias, and the sensitivity remains steadfast. However, the study was not without shortcomings. The follow-up periods were not consistently documented across the selected cases. Similarly, the duration of antifungal treatment reported varied and relied largely on the treating physician’s discretion. It should be noted that all the incorporated studies were retrospective in nature, which typically indicates a relatively lower research quality. The data in some subgroups was sparse, preventing us from drawing reliable conclusions from the analysis.

## Conclusion

This review offers the latest, most comprehensive snapshot of clinical presentations and mortality rates in mucor infected patients with hematological conditions. The collective mortality rate of hematological patients with mucormycosis stands at 61%. Notably, survival outcomes were better for patients receiving combined surgical and medical treatment and those with a singular infection site. Factors such as gender, breakthrough infections, pre-mucor infection use of mucor-active drugs, or multi-drug administration did not significantly impact the outcomes. The results of this meta-analysis underscore the need for future epidemiological studies on mucormycosis in hematological patients to better assess the role of various treatment regimens in reducing mortality risks.

## Data availability statement

The original contributions presented in the study are included in the article/Supplementary material, further inquiries can be directed to the corresponding author.

## Author contributions

MS: Conceptualization, Data curation, Writing – original draft. JW: Data curation, Methodology, Writing – review & editing. ML: Formal analysis, Methodology, Writing – review & editing. ZW: Data curation, Writing – review & editing.
