# Supplementary material for: The outcome and the risk factors of mucormycosis among patients with hematological diseases: a systematic and meta-analysis
Source: Front Med (Lausanne). 2023 Nov 30;10:1268840. doi: 10.3389/fmed.2023.1268840 (PMC10720036; doi:10.3389/fmed.2023.1268840)
Supplement: Supplementary file 1 [file Data_Sheet_1.docx]

**SUPPLEMENTARY APPENDIX**

Figure. S1. A sensitivity analysis of all the cases of mucormycosis among hematological patients.

Figure. S2. The risk difference (RD) of death in hematologic patients with mucormycosis in the male group (left side of the solid vertical line) versus the female group (right side of the solid vertical line). The individual RD is represented by grey squares, while a diamond indicates summary RD. The horizontal lines across the squares show the 95% confidence interval.

Figure. S3. The RD of death in hematologic patients with mucormycosis undergoing breakthrough infection (left side of the solid vertical line) versus those without breakthrough infection (right side of the solid vertical line). The individual RD is represented by grey squares, while a diamond indicates summary RD. The horizontal lines across the squares show the 95% confidence interval.

Figure. S4. The RD of death in hematologic patients with mucormycosis undergoing combined multi-drug therapy (left side of the solid vertical line) versus single-drug therapy (right side of the solid vertical line). The individual RD is represented by grey squares, while a diamond indicates summary RD. The horizontal lines across the squares show the 95% confidence interval.

Figure.S5. The RD of death in hematologic patients administrated mucor-unactive drugs (left side of the solid vertical line) versus mucor-active drugs (right side of the solid vertical line) prior to mucor infection. The individual RD is represented by grey squares, while a diamond indicates summary RD. The horizontal lines across the squares show the 95% confidence interval.

**Table S1: Summary of studies describing the mortality rate of mucormycosis among hematologic patients included in the systematic review and meta-analysis**

| ID | Author | Country | Published  Year | Duration | Age: median years（range） | Follow-up time | Death | Male | AML | ALL | Lymphoma | MDS | AA | MM | CLL | CML |
| --- | --- | --- | --- | --- | --- | --- | --- | --- | --- | --- | --- | --- | --- | --- | --- | --- |
| 1 | Matthieu Jestin (1) | France | 2021 | 2002-2018 | 38 (IQR 26-57) | 90d | 20/26 | 10/13 | 5/6 | 5/7 | 4/5 | - | 3/4 | - | - | - |
| 2 | Sarah Elitzur (2) | Israel | 2021 | 2009-2020 | 13.84 (8.9-19.2) | 261d | 7/10 | 3/4 | 2/4 | 4/5 | 1/1 | - | - | - | - | - |
| 3 | Hyeon-Jeong Lee (3) | Korea | 2020 | 2011-2018 | 50（23-77） | 12w | 7/26 | - | - | - | - | - | - | - | - | - |
| 4 | Johanna Claustre (4) | France | 2020 | 2008-2017 | - | - | 34/41 | - | 11/15 | 8/10 | 8/8 | 2/2 | - | 2/2 | - | - |
| 5 | Hyo-Ju Son (5) | Korea | 2020 | 2008-2019 | - | 90d | 20/39 | - | - | - | - | - | - | - | - | - |
| 6 | Ashrit Multani (6) | USA | 2019 | 2004-2018 | 53.1 (±15.6) | 1088d | 8/9 | 5/6 | 3/3 | 1/1 | 1/1 | - | - | - | - | - |
| 7 | Philipp Koehler (7) | Germany | 2019 | 2017-2018 | - | 365d | 8/11 | - | - | - | - | - | - | - | - | - |
| 8 | Abi Manesh (8) | India | 2019 | 2005-2015 | 39.64 mean | - | 16/28 | - | - | - | - | - | - | - | - | - |
| 9 | N. Klimko (9) | Russia | 2019 | 2007-2017 | 27（3-74） | 12w | 29/59 | - | - | - | - | - | - | - | - | - |
| 10 | Saeed El Zein(10) | USA | 2018 | 2008-2018 | 40.8 (±18.6) | 627d | 9/13 | 7/10 | 2/5 | 5/5 | 2/3 | - | - | - |  |  |
| 11 | Shobini Sivagnanam(11) | USA | 2017 | 2012-2015 | 57.5 (31-73) | 90d | 11/12 | 5/5 | 6/6 | 1/2 | 1/1 | 2/2 | - | 1/1 | - | 1/1 |
| 12 | Jesu's Guinea(12) | Spain | 2017 | 2007-2015 | 47 (4-61) | - | 5/9 | 4/7 | 0/2 | 1/2 | 4/4 | - | - | - | - | - |
| 13 | Reza Samarei(13) | Iran | 2017 | 2002-2016 | - | 200d | 11/12 | - | - | - | - | - | - | - | - | - |
| 14 | Andreas Kyvernitakis(14) | USA | 2016 | 1994-2014 | 53 (19-79) | 12w | 60/106 | 42/69 | - | - | - | - | - | - | - | - |
| 15 | Marjorie Roques(15) | France | 2016 | 2009-2015 | 59（33-75） | 365d | 6/15 | - | - | - | - | - | - | - | - | - |
| 16 | Arkadi Yakirevitch(16) | Israel | 2015 | 1998-2014 | 15（4-18） | 41m | 3/7 | - | 1/1 | 2/5 | 0/1 | - | - | - | - | - |
| 17 | Youngkyu Moon(17) | Korea | 2015 | 2011-2013 | 34.7 (±16.6) | 187d | 2/9 | - | - | - | - | - | - | - | - | - |
| 18 | L. Millon, R. (18) | France | 2015 | 2012-2014 | 48.0 (±18.3) | 12w | 25/33 | 18/21 | 6/11 | 4/6 | 8/9 | 4/4 | - | - | 2/2 | - |
| 19 | Ayesha Farooq(19) | USA | 2014 | 1999-2010 | 54 | 3m | 15/22 | - | - | - | - | - | - | - | - | - |
| 20 | Russell E. Lewis(20) | USA | 2014 | 2000-2012 | 57（16–76) | 4w | 28/75 | 15/50 | - | - | - | - | - | - | - | - |
| 21 | C. Legouge(21) | France | 2014 | 2003-2012 | 60 (32–74) | 90 | 15/16 | - | - | - | - | - | - | - | - | - |
| 22 | Aure'lie Phulpin-Weibel(22) | France | 2013 | 1991-2011 | 9.4（6m-20y） | 12m | 3/11 | 2/5 | 3/5 | 0/4 | 0/1 | - | - | - | - | - |
| 23 | Imad Abu El-Naaj(23) | Israel | 2013 | 2005-2011 | 40.5（15-75） | - | 5/6 | 2/3 | 2/2 | 0/1 | 1/1 | - | 1/1 | - | - | 1/1 |
| 24 | Lynne Strasfeld(24) | Poland | 2013 | 2005-2010 | 51.5 (±13.8) | 1y | 9/13 | 7/10 | 2/3 | 1/3 | - | 2/2 | - | 0/1 | - | 1/1 |
| 25 | Hyo-Jin Lee(25) | Korea | 2013 | 2007-2012 | 40.6 (±8.5) | 250d | 1/5 | 0/1 | 1/3 | - | - | - | 0/1 | 0/1 | - | - |
| 26 | Ozgur Tarkan(26) | Turkey | 2012 | 2006-2011 | 9（5-18） | 30m | 5/9 | 1/2 | 1/2 | 2/5 | 1/1 | - | 1/1 | - | - | - |
| 27 | Eloisa Llata(27) | USA | 2011 | 2003-2007 | 50（IQR36-66） | 14m | 5/11 | - | - | - | - | - | - | - | - | - |
| 28 | V. SAEGEMAN(28) | Belgium | 2010 | 2000-2007 | 56.4 (±12.5) | 25d | 9/9 | 6/6 | 3/3 | 1/1 | - | 3/3 | 1/1 | - | 1/1 | - |
| 29 | Juan Ambrosioni(29) | Switzerland | 2010 | 1989-2008 | 43.5（10-64） | - | 5/8 | 1/2 | - | - | - | - | - | - | - | - |
| 30 | Georgios Chamilos(30) | USA | 2008 | 1989-2006 | Death 53 (10–77)；Survival 46（6-60） | 12w | 46/70 | 27/45 | 17/28 | 7/10 | 7/9 | - | - | - | - | - |
| 31 | Hisham Wahba(31) | USA | 2008 | 2002-2007 | 48.0 (±18.3) | - | 4/6 | 3/4 | 2/3 | - | - | - | - | - | - | 1/1 |
| 32 | R. N. Greenberg(32) | USA | 2006 | 1999-2001 | 45.1 (±16.9) | - | 8/14 | 7/12 | 3/5 | 1/1 | 2/2 | 0/2 | 1/1 | - | 0/2 | 1/1 |
| 33 | Pagano, L. (33) | Italy | 2004 | 1987-2001 | mean 48(13-80) | - | 46/59 | 20/30 | - | - | - | - | - | - | - | - |
| 34 | R. Herbrecht(34) | France | 2001 | 1991-1994 | 35.4 (±17.5) | 112d | 5/12 | 4/8 | 2/4 | 0/2 | - | 1/1 | 1/1 | - | 1/1 | 0/3 |

(Continued)

| ID | Author | Proven | Probable | Possible  with positive PCR | Breakthrough IFI | Without breakthrough  IFI | Mucor-unactive IFI | Mucor-active IFI | Disseminate infection | Isolated infection | Single drug | Multi-drug | Medical-surgical therapy | Sole medical therapy |
| --- | --- | --- | --- | --- | --- | --- | --- | --- | --- | --- | --- | --- | --- | --- |
| 1 | Matthieu Jestin (1) | 12/14 | 2/5 | 6/7 | - | - | - | - | - | - | 12/14 | 8/12 | 6/8 | 14/18 |
| 2 | Sarah Elitzur (2) | 7/10 | - | - | 6/8 | 1/2 | 6/8 | - | 7/10 | - | 4/4 | 3/6 | 2/5 | 5/5 |
| 3 | Hyeon-Jeong Lee (3) | - | - | - | 3/6 | 3/20 | - | - | - | - | - | - | 2/20 | 4/6 |
| 4 | Johanna Claustre (4) | - | - | - | - | - | - | - | - | - | - | - | - | - |
| 5 | Hyo-Ju Son (5) | - | - | - | - | - | - | - | - | - | - | - | - | - |
| 6 | Ashrit Multani (6) | - | - | - | - | - | - | - | 2/2 | 6/7 | - | - | 8/9 | - |
| 7 | Philipp Koehler (7) | - | - | - | - | - | - | - | - | - | - | - | - | - |
| 8 | Abi Manesh (8) | 16/28 | - | - | - | - | - | - | - | - | - | - | - | - |
| 9 | N. Klimko (9) | - | - | - | - | - | - | - | - | - | - | - | - | - |
| 10 | Saeed El Zein(10) | 3/5 | 6/8 | - | 7/9 | 2/4 | 8/10 | 2/2 | 1/1 | 8/12 | 3/7 | 6/6 | 5/9 | 4/4 |
| 11 | Shobini Sivagnanam(11) | 6/7 | - | 5/5 | 10/11 | 1/1 | 11/12 | 2/2 | 3/3 | 8/9 | 4/5 | 3/3 | 3/4 | 5/5 |
| 12 | Jesu's Guinea(12) | 5/5 | - | - | 4/7 | 1/2 | 3/4 | 0/2 | 1/1 | 4/8 | 2/2 | 3//7 | - | - |
| 13 | Reza Samarei(13) | - | - | - | - | - | - | - | - | - | - | - | - | - |
| 14 | Andreas Kyvernitakis(14) | 40/74 | 20/32 | - | 53/94 | 7/12 | 51/92 | 2/2 | 14/20 | 43/72 | 26/47 | 27/59 | 18/40 | 42/66 |
| 15 | Marjorie Roques(15) | - | - | - | - | - | - | - | - | 6/15 | - | - | - | - |
| 16 | Arkadi Yakirevitch(16) | - | - | - | - | - | - | - | - | - | 2/5 | 1/2 | 3/7 | - |
| 17 | Youngkyu Moon(17) | - | - | - | - | - | - | - | - | - | - | - | 2/9 | - |
| 18 | L. Millon, R. (18) | 12/18 | 13/15 | - | - | - | - | - | 7/10 | 18/23 | - | - | - | - |
| 19 | Ayesha Farooq(19) | - | - | - | - | - | - | - | - | - | - | - | - | - |
| 20 | Russell E. Lewis(20) | - | - | - | 21/56 | 7/19 | 18/40 | - | 3/12 | 25/63 | - | - | - | - |
| 21 | C. Legouge(21) | 10/16 | - | - | - | - | - | - | - | - | 2/7 | 1/4 | 6/6 | 4/10 |
| 22 | Aure'lie Phulpin-Weibel(22) | - | - | - | 3/5 | 0/6 | 6/8 | 0/1 | 1/3 | 2/8 | - | - | 1/7 | 2/4 |
| 23 | Imad Abu El-Naaj(23) | - | - | - | - | - | - | - | - | - | - | 5/6 | 5/6 | - |
| 24 | Lynne Strasfeld(24) | - | - | - | 9/13 | - | - | - | - | - | 6/8 | 3/5 | 3/6 | 6/7 |
| 25 | Hyo-Jin Lee(25) | - | - | - | - | - | - | - | 1/3 | 0/2 | - | 1/5 | 1/5 | - |
| 26 | Ozgur Tarkan(26) | - | - | - | - | - | - | - | - | - | - | - | 5/9 | - |
| 27 | Eloisa Llata(27) | - | - | - | - | - | - | - | - | - | - | - | - | - |
| 28 | V. SAEGEMAN(28) | - | - | - | - | - | - | - | 1/1 | 8/8 | 7/7 | - | 1/1 | 6/6 |
| 29 | Juan Ambrosioni(29) | - | - | - | 4/7 | 1/1 | 3/4 | 1/1 | 3/5 | 2/3 | 1/1 | 1/4 | 1/2 | 4/6 |
| 30 | Georgios Chamilos(30) | 35/45 | 11/25 | - | 27/41 | 19/29 | - | - | 10/11 | 36/59 | 46/70 | 16/36 | 13/22 | 33/48 |
| 31 | Hisham Wahba(31) | - | - | - | - | - | - | - | - | - | - | - | - | - |
| 32 | R. N. Greenberg(32) | - | - | - | - | - | - | - | 4/4 | 4/10 | - | - | - | - |
| 33 | Pagano, L. (33) | - | - | - | 36/47 | 10/12 | - | - | - | - | - | - | - | - |
| 34 | R. Herbrecht(34) | - | - | - | 5/12 | - | - | - | 2/4 | 3/8 | - | - | - | - |

IQR, interquartile range; PCR, Polymerase Chain Reaction; d, day; w, week; m, month; y, year; IFI, invasive fungal infection; -, Not available; AML, acute myeloid leukemia; ALL, acute lymphocytic leukemia; MDS, myelodysplasia syndrome; AA, aplastic anemia; MM, multiple myeloma; CLL, chronic lymphocytic leukemia; CML, chronic myeloid leukemia.

**Table S2:** **Newcastle Ottawa score to assess the quality of the included studies for the outcome of mucormycosis among hematologic patients.**

| ID | Author/Country | year | Selection | Comparability | Outcome | Overall score |
| --- | --- | --- | --- | --- | --- | --- |
| 1 | Matthieu Jestin (1) | 2021 | 4 | 2 | 3 | 9 |
| 2 | Sarah Elitzur (2) | 2021 | 3 | - | 3 | 6 |
| 3 | Hyeon-Jeong Lee (3) | 2020 | 4 | 2 | 2 | 8 |
| 4 | Johanna Claustre (4) | 2020 | 4 | 2 | 2 | 8 |
| 5 | Hyo-Ju Son (5) | 2020 | 4 | 2 | 2 | 8 |
| 6 | Ashrit Multani (6) | 2019 | 3 | - | 3 | 6 |
| 7 | Philipp Koehler (7) | 2019 | 3 | - | 3 | 6 |
| 8 | Abi Manesh (8) | 2019 | 3 | - | 2 | 5 |
| 9 | N. Klimko (9) | 2019 | 3 | - | 3 | 6 |
| 10 | Saeed El Zein (10) | 2018 | 3 | - | 3 | 6 |
| 11 | Shobini Sivagnanam (11) | 2017 | 3 | - | 3 | 6 |
| 12 | Jesu ́ s Guinea (12) | 2017 | 3 | - | 2 | 5 |
| 13 | Reza Samarei (13) | 2017 | 4 | - | 3 | 7 |
| 14 | Andreas Kyvernitakis (14) | 2016 | 4 | 2 | 3 | 9 |
| 15 | Marjorie Roques (15) | 2016 | 4 | 0 | 2 | 6 |
| 16 | Arkadi Yakirevitch (16) | 2015 | 3 | - | 3 | 6 |
| 17 | Youngkyu Moon (17) | 2015 | 3 | - | 3 | 6 |
| 18 | L. Millon, R. (18) | 2015 | 3 | - | 3 | 6 |
| 19 | Ayesha Farooq (19) | 2014 | 3 | - | 2 | 5 |
| 20 | Russell E. Lewis (20) | 2014 | 4 | 2 | 2 | 8 |
| 21 | C. Legouge (21) | 2014 | 3 | - | 3 | 6 |
| 22 | Aure ́ lie Phulpin-Weibel (22) | 2013 | 3 | - | 3 | 6 |
| 23 | Imad Abu El-Naaj (23) | 2013 | 3 | - | 3 | 6 |
| 24 | Lynne Strasfeld (24) | 2013 | 3 | - | 3 | 6 |
| 25 | Hyo-Jin Lee (25) | 2013 | 3 | - | 3 | 6 |
| 26 | Ozgur Tarkan (26) | 2012 | 3 | - | 3 | 6 |
| 27 | Eloisa Llata (27) | 2011 | 3 | - | 3 | 6 |
| 28 | V. SAEGEMAN (28) | 2010 | 3 | - | 3 | 6 |
| 29 | Juan Ambrosioni (29) | 2010 | 3 | - | 2 | 5 |
| 30 | Georgios Chamilos (30) | 2008 | 4 | 1 | 3 | 8 |
| 31 | Hisham Wahba (31) | 2008 | 3 | - | 3 | 6 |
| 32 | R. N. Greenberg (32) | 2006 | 3 | - | 3 | 6 |
| 33 | Pagano, L. (33) | 2004 | 3 | - | 2 | 5 |
| 34 | R. Herbrecht (34) | 2001 | 3 | - | 3 | 6 |

**Reference**

1. Jestin M, Azoulay E, Pene F, Bruneel F, Mayaux J, Murgier M, et al. Poor outcome associated with mucormycosis in critically ill hematological patients: results of a multicenter study. Annals of Intensive Care. 2021;11(1):8.

2. Elitzur S, Fischer S, Arad-Cohen N, Barg A, Ben-Harosh M, Danino D, et al. Disseminated Mucormycosis in Immunocompromised Children: Are New Antifungal Agents Making a Difference? A Multicenter Retrospective Study. J Fungi. 2021;7(3):13.

3. Lee HJ, Cho SY, Lee DG, Park C, Chun HS, Park YJ. Characteristics and risk factors for mortality of invasive non-Aspergillus mould infections in patients with haematologic diseases: A single-centre 7-year cohort study. Mycoses. 2020;63(3):257-64.

4. Claustre J, Larcher R, Jouve T, Truche AS, Nseir S, Cadiet J, et al. Mucormycosis in intensive care unit: surgery is a major prognostic factor in patients with hematological malignancy. Ann Intensive Care. 2020;10(1):74.

5. Son HJ, Song JS, Choi S, Jung J, Kim MJ, Chong YP, et al. Risk factors for mortality in patients with pulmonary mucormycosis. Mycoses. 2020;63(7):729-36.

6. Multani A, Reveron-Thornton R, Garvert DW, Gomez CA, Montoya JG, Lui NS. Cut it out! Thoracic surgeon's approach to pulmonary mucormycosis and the role of surgical resection in survival. Mycoses. 2019;62(10):893-907.

7. Koehler P, Mellinghoff SC, Lagrou K, Alanio A, Arenz D, Hoenigl M, et al. Development and validation of the European QUALity (EQUAL) score for mucormycosis management in haematology. J Antimicrob Chemother. 2019;74(6):1704-12.

8. Manesh A, Rupali P, Sullivan MO, Mohanraj P, Rupa V, George B, et al. Mucormycosis-A clinicoepidemiological review of cases over 10 years. Mycoses. 2019;62(4):391-8.

9. Klimko N, Khostelidi S, Shadrivova O, Volkova A, Popova M, Uspenskaya O, et al. Contrasts between mucormycosis and aspergillosis in oncohematological patients. Med Mycol. 2019;57(Supplement_2):S138-s44.

10. El Zein S, El-Cheikh J, El Zakhem A, Ibrahim D, Bazarbachi A, Kanj SS. Mucormycosis in hospitalized patients at a tertiary care center in Lebanon: a case series. Infection. 2018;46(6):811-21.

11. Sivagnanam S, Sengupta DJ, Hoogestraat D, Jain R, Stednick Z, Fredricks DN, et al. Seasonal clustering of sinopulmonary mucormycosis in patients with hematologic malignancies at a large comprehensive cancer center. Antimicrob Resist Infect Control. 2017;6:123.

12. Guinea J, Escribano P, Vena A, Muñoz P, Martínez-Jiménez MDC, Padilla B, et al. Increasing incidence of mucormycosis in a large Spanish hospital from 2007 to 2015: Epidemiology and microbiological characterization of the isolates. PLoS One. 2017;12(6):e0179136.

13. Samarei R, Gharebaghi N, Zayer S. Evaluation of 30 cases of mucormycosis at a university hospital in Iran. Mycoses. 2017;60(7):426-32.

14. Kyvernitakis A, Torres HA, Jiang Y, Chamilos G, Lewis RE, Kontoyiannis DP. Initial use of combination treatment does not impact survival of 106 patients with haematologic malignancies and mucormycosis: a propensity score analysis. Clin Microbiol Infect. 2016;22(9):811.e1-.e8.

15. Roques M, Chretien ML, Favennec C, Lafon I, Ferrant E, Legouge C, et al. Evolution of procalcitonin, C-reactive protein and fibrinogen levels in neutropenic leukaemia patients with invasive pulmonary aspergillosis or mucormycosis. Mycoses. 2016;59(6):383-90.

16. Yakirevitch A, Barg AA, Bedrin L, Primov-Fever A, Wolf M, Migirov L. Acute Invasive Fungal Rhinosinusitis in Children With Hematologic Malignancies: Outcome of Surgical Treatment. Pediatr Hematol Oncol. 2015;32(8):568-75.

17. Moon Y, Park JK, Sung SW. Surgery for localized pulmonary mycotic infections in patients with hematopoietic disorder. J Cardiothorac Surg. 2015;10:7.

18. Millon L, Herbrecht R, Grenouillet F, Morio F, Alanio A, Letscher-Bru V, et al. Early diagnosis and monitoring of mucormycosis by detection of circulating DNA in serum: retrospective analysis of 44 cases collected through the French Surveillance Network of Invasive Fungal Infections (RESSIF). Clin Microbiol Infect. 2015;22(9):810 e1- e8.

19. Farooq A, Alrabaa S, Quilitz R, Yacoub A, Maroon E, Fulp W, et al. Comparison of Clinical and Radiological Features of Aspergillus, Zygomycosis, and Fusarium Pneumonia in Neutropenic Patients. Infectious Diseases in Clinical Practice. 2014;22(5):288-93.

20. Lewis RE, Georgiadou SP, Sampsonas F, Chamilos G, Kontoyiannis DP. Risk factors for early mortality in haematological malignancy patients with pulmonary mucormycosis. Mycoses. 2014;57(1):49-55.

21. Legouge C, Caillot D, Chrétien ML, Lafon I, Ferrant E, Audia S, et al. The reversed halo sign: pathognomonic pattern of pulmonary mucormycosis in leukemic patients with neutropenia? Clin Infect Dis. 2014;58(5):672-8.

22. Phulpin-Weibel A, Rivier A, Leblanc T, Bertrand Y, Chastagner P. Focus on invasive mucormycosis in paediatric haematology oncology patients: a series of 11 cases. Mycoses. 2013;56(3):236-40.

23. Abu El-Naaj I, Leiser Y, Wolff A, Peled M. The surgical management of rhinocerebral mucormycosis. J Craniomaxillofac Surg. 2013;41(4):291-5.

24. Strasfeld L, Espinosa-Aguilar L, Gajewski JL, Stenzel P, Pimentel A, Mater E, et al. Emergence of Cunninghamella as a pathogenic invasive mold infection in allogeneic transplant recipients. Clin Lymphoma Myeloma Leuk. 2013;13(5):622-8.

25. Lee HJ, Kwon JC, Kim SH, Choi SM, Lee DG, Park SH, et al. Posaconazole treatment in Korea: single-center experience over 5 years. Yonsei Med J. 2013;54(5):1234-40.

26. Tarkan O, Karagün B, Ozdemir S, Tuncer U, Sürmelioğlu O, Cekiç E, et al. Endonasal treatment of acute invasive fungal rhinosinusitis in immunocompromised pediatric hematology-oncology patients. Int J Pediatr Otorhinolaryngol. 2012;76(10):1458-64.

27. Llata E, Blossom DB, Khoury HJ, Rao CY, Wannemuehler KA, Noble-Wang J, et al. A cluster of mucormycosis infections in hematology patients: challenges in investigation and control of invasive mold infections in high-risk patient populations. Diagn Microbiol Infect Dis. 2011;71(1):72-80.

28. Saegeman V, Maertens J, Ectors N, Meersseman W, Lagrou K. Epidemiology of mucormycosis: review of 18 cases in a tertiary care hospital. Medical Mycology. 2010;48(2):245-54.

29. Ambrosioni J, Bouchuiguir-Wafa K, Garbino J. Emerging invasive zygomycosis in a tertiary care center: epidemiology and associated risk factors. Int J Infect Dis. 2010;14 Suppl 3:e100-3.

30. Chamilos G, Lewis RE, Kontoyiannis DP. Delaying amphotericin B-based frontline therapy significantly increases mortality among patients with hematologic malignancy who have zygomycosis. Clin Infect Dis. 2008;47(4):503-9.

31. Wahba H, Truong MT, Lei X, Kontoyiannis DP, Marom EM. Reversed halo sign in invasive pulmonary fungal infections. Clin Infect Dis. 2008;46(11):1733-7.

32. Greenberg RN, Mullane K, van Burik JA, Raad I, Abzug MJ, Anstead G, et al. Posaconazole as salvage therapy for zygomycosis. Antimicrob Agents Chemother. 2006;50(1):126-33.

33. Pagano L, Offidani M, Fianchi L, Nosari A, Candoni A, Piccardi M, et al. Mucormycosis in hematologic patients. Haematologica. 2004;89(2):207-14.

34. Herbrecht R, Letscher-Bru V, Bowden RA, Kusne S, Anaissie EJ, Graybill JR, et al. Treatment of 21 cases of invasive mucormycosis with amphotericin B colloidal dispersion. Eur J Clin Microbiol Infect Dis. 2001;20(7):460-6.
